# Different Loci of Semantic Interference in Picture Naming vs. Word-Picture Matching Tasks

**DOI:** 10.3389/fpsyg.2016.00710

**Published:** 2016-05-13

**Authors:** Denise Y. Harvey, Tatiana T. Schnur

**Affiliations:** ^1^Department of Neurology, University of PennsylvaniaPhiladelphia, PA, USA; ^2^Moss Rehabilitation Research InstituteElkins Park, PA, USA; ^3^Baylor College of Medicine, Department of NeurosurgeryHouston, TX, USA

**Keywords:** semantic interference, lexical access, semantic access, generalization of interference, lexical frequency

## Abstract

Naming pictures and matching words to pictures belonging to the same semantic category impairs performance relative to when stimuli come from different semantic categories (i.e., semantic interference). Despite similar semantic interference phenomena in both picture naming and word-picture matching tasks, the locus of interference has been attributed to different levels of the language system – lexical in naming and semantic in word-picture matching. Although both tasks involve access to shared semantic representations, the extent to which interference originates and/or has its locus at a shared level remains unclear, as these effects are often investigated in isolation. We manipulated semantic context in cyclical picture naming and word-picture matching tasks, and tested whether factors tapping semantic-level (generalization of interference to novel category items) and lexical-level processes (interactions with lexical frequency) affected the magnitude of interference, while also assessing whether interference occurs at a shared processing level(s) (transfer of interference across tasks). We found that semantic interference in naming was sensitive to both semantic- and lexical-level processes (i.e., larger interference for novel vs. old and low- vs. high-frequency stimuli), consistent with a semantically mediated lexical locus. Interference in word-picture matching exhibited stable interference for old and novel stimuli and did not interact with lexical frequency. Further, interference transferred from word-picture matching to naming. Together, these experiments provide evidence to suggest that semantic interference in both tasks originates at a shared processing stage (presumably at the semantic level), but that it exerts its effect at different loci when naming pictures vs. matching words to pictures.

## Introduction

Accessing words (lexical representations) and meanings (semantic representations) from the same vs. different categories can interfere with future access from the category. For example, patients with aphasia due to stroke tend to make semantic errors when naming pictures and/or matching words to pictures in the context of semantically related words (e.g., Schnur et al., 2006; Biegler et al., 2008; Harvey and Schnur, 2015). Moreover, naming pictures (e.g., Kroll and Stewart, 1994) or matching words to pictures (Campanella and Shallice, 2011) belonging to the same semantic category has a detrimental effect on healthy participants’ performance, known as semantic interference. That both picture naming and word-picture matching performance is sensitive to semantic contexts demonstrates that both tasks are semantically mediated (see Belke, 2013). However, because semantic interference in picture naming and word-picture matching tasks is usually investigated separately, this has led to different conclusions about the locus of interference in each task. In picture naming, evidence suggests that interference arises when mapping from semantic to lexical representations (hereafter, lexical locus; Levelt et al., 1999; Howard et al., 2006; Oppenheim et al., 2010), whereas in word-picture matching tasks evidence suggests that interference arises within the semantic system itself (Warrington and McCarthy, 1983, 1987; Warrington and Cipolotti, 1996; Forde and Humphreys, 1997, 2007; Gotts and Plaut, 2002; Campanella and Shallice, 2011). While semantic interference in picture naming tasks has been largely explored in healthy subjects, semantic interference in word-picture matching tasks is less often reported in the healthy population (cf. Biegler et al., 2008; Campanella and Shallice, 2011; Wei and Schnur, 2016). Here, we investigated the locus of semantic interference in naming and word-picture matching by testing in healthy participants whether interference was sensitive to semantic and lexical factors and transferred between the two tasks. Finding that interference is affected by the same factors and/or transfers across the two tasks can elucidate the extent to which processes governing access to semantic and lexical representations operate similarly across the two tasks. In turn, this work informs theories of lexical-semantic access, providing clues about the organization of the language system as a whole.

In both picture naming and word-picture matching tasks, repeatedly accessing semantically related stimuli has a negative effect on performance. For example, participants are slower to name pictures or match words to pictures when trials depict items belonging to the same categories (related context: e.g., CAT, DOG, BEAR, and COW) vs. different categories (unrelated context: e.g., CAT, TRAIN, SHIRT, and DESK)^1^ (i.e., blocked naming and word-picture matching tasks; e.g., Damian et al., 2001; Damian and Als, 2005; Campanella and Shallice, 2011). Interference is thought to occur because activating the semantic system to produce a target word (i.e., “dog”) or access a word’s meaning (i.e., DOG) results in the co-activation of related words and meanings (e.g., “cat” and CAT) due to the high degree of semantic feature overlap amongst members of the same category (e.g., Collins and Loftus, 1975; see also Vigliocco et al., 2002; Forde and Humphreys, 2007). This is evidenced by the findings of graded semantic interference effects in both tasks (i.e., larger interference for semantically close vs. distant category members; naming: Vigliocco et al., 2002; Navarrete et al., 2012; word-picture matching: Crutch and Warrington, 2005, Experiment 1; Warrington and Cipolotti, 1996, Experiment 5). That naming and word-picture matching are sensitive to semantic contexts demonstrates that interference in both tasks originates at the semantic level.

However, the locus of semantic interference in each task is thought to differ. By most accounts, semantic interference in naming exerts its effects at the lexical level (e.g., Howard et al., 2006; Oppenheim et al., 2010; see also Damian and Als, 2005; cf. Levelt et al., 1999; Damian et al., 2001), whereas semantic interference in word-picture matching exerts its effects at the semantic level (e.g., Forde and Humphreys, 1997, 2007; Campanella and Shallice, 2011). Computational models of semantic interference in naming (Howard et al., 2006; Oppenheim et al., 2010; see also Roelofs, 1992) assume that naming a picture (i.e., DOG) activates its lexical representation (i.e., “dog”) and those sharing semantic features with the target (e.g., “cat”) to a greater extent than those that do not share semantic features with the target (e.g., “shoe”). Producing the word “dog” increases its lexical representation’s activation level, which negatively affects the subsequent selection of same-category lexical representations (e.g., “cat”). Accordingly, theories of semantic interference in naming (e.g., Howard et al., 2006; Oppenheim et al., 2010) assume that shared activation at the semantic level causes interference that exerts its effects at a lexical level. Theories of semantic interference in word-picture matching assume that activating the meaning of a given word (i.e., “dog”) also activates related word meanings (e.g., CAT), which interfere with the ability to distinguish between same-category meanings on subsequent trials (Forde and Humphreys, 1997, 2007; see also Warrington and Cipolotti, 1996; Gotts and Plaut, 2002). Thus, semantic interference in naming originates at the semantic level, but has a lexical-level locus (see also Belke, 2013), whereas semantic interference in word-picture matching both originates and has its locus at the semantic level.

That semantic contexts are thought to interfere with word-picture matching performance at a semantic level seemingly contradicts the generally accepted view that semantic contexts facilitate performance on tasks requiring semantic but not lexical access for spoken output (Bajo, 1988; Belke, 2013). For example, semantic relationships facilitate the recognition of words preceded by a semantically related prime word (i.e., lexical decision task; e.g., McRae and Boisvert, 1998) and the categorization of pictured objects based on the direction (i.e., left or right) they face (i.e., orientation judgment task; Damian et al., 2001)^2^ or based on their superordinate category (i.e., man-made or natural) membership (i.e., semantic classification task; Belke, 2013; see also Vitkovitch and Humphreys, 1991). However, tasks argued to tap semantic level facilitatory processes differ in a number of respects with those eliciting semantic interference. In the lexical decision task, “…co-activation of other words would not be costly because the task only requires participants to decide whether the presented string is a word or not…” (Vigliocco et al., 2004, p. 468) but not whether the word refers to a specific meaning. Further, judging the orientation of a pictured object (i.e., tip of a shoe) in terms of which direction it faces relies more on decoding the visual properties of the object (e.g., Humphreys et al., 1988, 1995) and not necessarily the semantic features corresponding to the object (see Belke, 2013). Lastly, the semantic classification task while likely requiring access to semantic information, does not necessitate accessing fine-grained semantic level distinguishing information, as all members of a semantic category are consistent with the classification of man-made or natural. By contrast, matching a word to its corresponding picture necessitates making fine-grained semantic decisions about the set of semantic features associated with that particular word, which in some ways is like naming a picture, as picture naming necessitates the selection of a word based on the set of semantic features that distinguish the target lexical representation from co-activated, semantically related, lexical representations (see Wei and Schnur, 2016 for a similar discussion). Thus, the assumption that semantic contexts facilitate processing at the semantic level may be an artifact of the types of tasks used to tap semantic-level processes (see Chen and Mirman, 2012 for a similar argument).

Does semantic interference occur in the healthy semantic system when discriminating a target from related meanings? Evidence of semantic interference in word-picture matching almost exclusively comes from neuropsychological studies of patients with aphasia secondary to stroke (cf. Biegler et al., 2008; Campanella and Shallice, 2011; Wei and Schnur, 2016). Consequently, the extent to which the healthy semantic system operates similarly when accessing words and meanings is not well understood. To our knowledge, only a few studies have investigated semantic interference in healthy younger adults’ word-picture matching performance, demonstrating that semantic interference occurs in tasks tapping semantic-level processes (Campanella and Shallice, 2011; Mirman and Graziano, 2012; Wei and Schnur, 2016; see Biegler et al., 2008 for evidence of semantic interference in healthy older adults’ word-picture matching performance). What remains unclear is whether the semantic context effects observed in word-picture matching occur due to the same processes that create interference in naming.

### The Current Research

The main goal of this research was to investigate whether semantic interference in naming and word-picture matching originate and/or exert their effects at a shared processing level(s). Accordingly, we explored how factors that tap semantic and lexical processing affect semantic interference in each task, and whether semantic interference at a shared processing level(s) allows for the effect to transfer across tasks.

**FIGURE 1:**
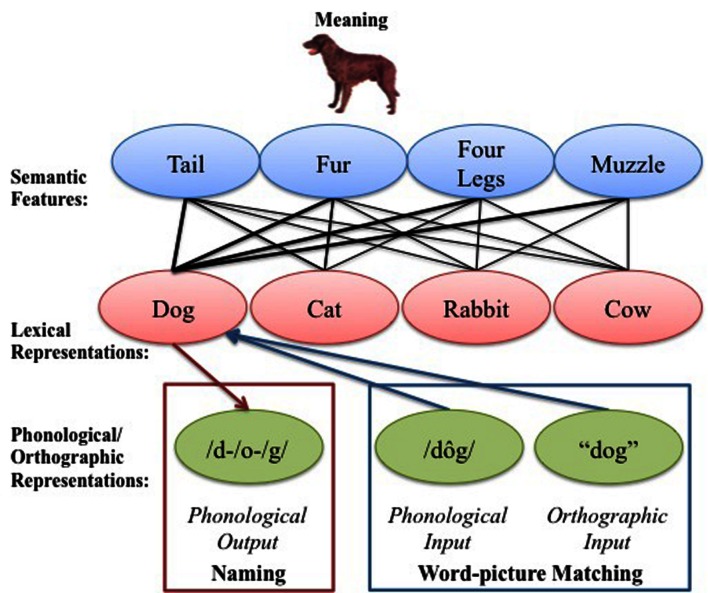
**The basic structure of the language system.** It is hypothesized that naming and matching words to pictures require access to shared semantic and lexical level representations, but the output vs. input levels of representation differ across the tasks (cf. Caramazza, 1997; Levelt et al., 1999).

Here, we used cyclical variants of the blocked naming and word-picture matching tasks, where subjects name pictures or match words to pictures in related vs. unrelated contexts, and target items repeat multiple times (cycles) in different orders (e.g., Kroll and Stewart, 1994; Damian, et al., 2001; Campanella and Shallice, 2011; see also Wei and Schnur, 2016). Whether assuming a lexical- or semantic-level locus, interference is thought to emerge with repetition because competition increases with repeated access to same- vs. different-category items (e.g., Forde and Humphreys, 1995, 2007; Belke et al., 2005b; cf. Navarrete et al., 2014 for an alternative account in naming). We hypothesize that because both picture naming and word-picture matching tasks require mapping between shared lexical and semantic representational levels (see **Figure 1**; reviewed in Howard, 1995; Levelt, 1999; cf. Caramazza, 1997), it suggests a shared origin and/or locus of interference in the two tasks. Specifically, in both tasks it is necessary to access the semantic features corresponding to the target representation (picture or word form) – a process that results in the co-activation of related representations. However, because the order with which lexical and semantic representations are activated occurs in reverse in the two tasks (semantic-to-lexical in naming and vice versa in word-picture matching), the level at which co-activated representations interfere with performance is thought to differ. Consequently, it remains an open question as to whether semantic interference in the two tasks is a reflection of the same underlying phenomena occurring at shared semantic and/or lexical representational levels.

#### Origin of Interference

Because interference is assumed to originate at the semantic level in both picture naming and word-picture matching, it is predicted to generalize to novel category members (Forde and Humphreys, 1995; Belke et al., 2005b) and transfer across tasks (Belke, 2013). In naming, shared activation at the semantic level gives rise to the co-activation of related lexical representations (Howard et al., 2006; Oppenheim et al., 2010), resulting in the accumulation of semantic interference for both previously named and novel category members (e.g., Belke et al., 2005b). Similarly, in word-picture matching, accessing semantically related word meanings in succession renders disambiguating both previously accessed and novel word meanings belonging to the same category more difficult (e.g., Forde and Humphreys, 1995, 2007). Moreover, a shared semantic-level origin of interference in naming and word-picture matching predicts that interference will transfer across tasks. For example, if accessing the semantic system in word-picture matching results in the co-activation of both related semantic and lexical representations, then this should interfere with subsequent naming of novel same-category pictures. Thus, interference originating at the semantic level predicts both generalization of interference within each task, and transfer of interference across tasks.

To our knowledge, there are only two studies that have demonstrated interference generalization using the blocked-cyclic paradigms: one in healthy subjects’ picture naming performance (Belke et al., 2005b, Experiment 3) and the other in an aphasic patient’s comprehension performance (patient J.M.; Forde and Humphreys, 1995, Experiment 12). Belke et al. (2005b) examined whether interference generalized to cycles of naming novel items semantically related to those named previously. They found that semantic interference emerged after the first cycle and remained unchanged across subsequent cycles of both previously named and novel pictures. Forde and Humphreys (1995) found that in comprehension (i.e., auditory-to-written word matching), the interference effect was larger for the first cycle of novel words relative to the first cycle of semantically related “old” words. Thus, generalization of semantic interference has been quantified in two different ways. Belke et al. (2005b) defined generalization as an unchanging effect (once it emerges after cycle 1) across old and novel items (cycles 2–8), whereas Forde and Humphreys defined generalization as larger interference for the first cycle of novel compared to the first cycle of old items. In the experiments reported here, we assessed generalization as an increase in interference for novel compared to old items collapsed across cycles because both characterizations (i.e., no interference at cycle 1 followed by unchanging interference for cycles 2–8, Belke et al., 2005b and larger interference for the first cycle of novel vs. the first cycle of old items, Forde and Humphreys, 1995) should when averaged across cycles yield larger interference for novel vs. old items. The first goal of this study was to replicate and extend the findings of interference generalization obtained in previous studies using blocked-cyclic naming and word-picture matching tasks to demonstrate that interference in both tasks originates at the semantic level.

#### Locus of Interference

Because theories of semantic interference in naming and word-picture matching tasks assume different semantic interference loci (e.g., Forde and Humphreys, 2007; Oppenheim et al., 2010), this generates the prediction that lexical frequency, a factor thought to exert its effects primarily at the name retrieval stage (e.g., Vitkovitch and Humphreys, 1991),^3^ should affect semantic interference in naming but not word-picture matching as word-picture matching does not require access to lexical representations for spoken output (see Campanella and Shallice, 2011 for discussion). That subjects name pictures depicting high-frequency words faster than those depicting low-frequency words (e.g., Oldfield and Wingfield, 1965) and recognize high- vs. low-frequency words faster in word recognition tasks such as the lexical decision task (e.g., Scarborough et al., 1977; Balota and Chumbley, 1985) indicates that high- vs. low-frequency lexical representations have increased activation levels (Morton, 1969; McClelland and Rumelhart, 1981; Dell, 1986; Seidenberg and McClelland, 1989; Plaut et al., 1996; Caramazza, 1997; Barry et al., 2001; Kittredge et al., 2008), rendering them more available for selection in naming and identification in word recognition. Thus, a lexical, but not semantic, locus of interference predicts that interference will be affected by the lexical frequency of semantically related words when naming pictures, but not when matching words to pictures.

Although lexical frequency is predicted to interact with semantic interference in naming but not word-picture matching, previous studies investigating these factors provide equivocal results. To our knowledge, there is only one study that examined the effect of lexical frequency on response times (RTs) in blocked-cyclic naming and although there was an overall effect of lexical frequency on naming, it did not interact with semantic interference (Santesteban et al., 2006). Campanella and Shallice (2011) manipulated semantic context (close vs. distant) and lexical frequency (high- vs. low-frequency) in a non-cyclical word-picture matching task, and found that healthy subjects were slower and less accurate in the semantically close, low-frequency condition compared to all other conditions (i.e., semantically close, high-frequency, semantically distant high-frequency, and semantically distant, low-frequency; Experiment 1). Experiment 2 used a cyclical variant of the task, testing only those items that gave rise to the largest interference effects in Experiment 1 (i.e., semantically close, low-frequency words), and found that interference increased across cycles of repeated word-picture matching. That semantic interference was numerically larger for low- compared to high-frequency words (Experiment 1) contradicts a semantic locus of interference, suggesting instead that interference in word-picture matching has a lexical locus. Thus, the second aim of this study was to test whether lexical frequency interacts with semantic interference in naming and/or word-picture matching to determine whether or not the locus of interference is shared across the two tasks.

Lastly, it remains an open question whether or not semantic interference observed in picture naming and word-picture matching arises due to the same or partially overlapping processing stages. To our knowledge, no one has tested whether semantic interference transfers across the two tasks. However, previous work has examined interference transfer to and from different levels of the language system, but the evidence here is mixed. While Navarrete et al. (2010) found that interference transferred from a task tapping semantically mediated lexical retrieval (i.e., picture + determiner naming) to one requiring lexical retrieval without semantic mediation (i.e., word + determiner naming) but not vice versa (Experiment 3), Belke (2013) did not replicate this finding (Experiment 4). Moreover, Belke (2013) demonstrated that picture naming affected subsequent semantic classification (i.e., man-made or natural) of categorically related objects but not vice versa (Experiment 5), which conflicts with previous evidence that semantic classification affects the subsequent naming of categorically related pictures (Vitkovitch and Humphreys, 1991, Experiment 2). Consequently, the extent to which interference transfers across tasks tapping shared representational levels remains unclear. Thus, the third goal of this study was to investigate whether a shared origin and/or locus of interference exists, as evidenced by increased semantic interference (and thus transfer) when performing the naming (or word-picture matching) task on novel items categorically related to those which appeared previously in the word-picture matching (or naming) task. If the origin *and* locus of interference is shared in naming and word-picture matching, then interference will be sensitive to both semantic and lexical factors within each task (Experiments 1 and 2) and transfer across tasks (Experiment 3). However, if interference has a shared origin but different loci, then interference will generalize within tasks and transfer across tasks, but only inference in naming will interact with lexical frequency.

## Materials and Methods

### Participants

There were 94 participants total. Thirty-one participated in Experiment 1 [15 female, 16 male; mean (and range) age: 19 years (18–21)], 20 participated in Experiment 2 [12 female, 8 male; mean (and range) age: 19 years (18–22)], and 43 in Experiment 3 [25 female, 18 male; mean (and range) age: 19 years (18–22)]. Data from four participants who took part in Experiment 1 were excluded: two due to experimenter error and two due to equipment error. All were native English speakers with normal or corrected to normal vision attending Rice University, and received course credit for their participation. Informed consent in accordance with the IRB at Rice University was obtained from each participant.

### Materials and Design

Stimuli were 64 colored pictures of familiar objects belonging to eight semantic categories. Pictures were taken from the Bank of Standardized Stimuli (BOSS; Brodeur et al., 2010) and another image database (Viggiano et al., 2004), and scaled to 400 pixels × 400 pixels. Within each category, pictures consisted of all high- or low-frequency names, and were selected to minimize differences in other factors known to correlate with lexical frequency, such as familiarity and imageability (e.g., Morrison et al., 1997). Measures of lexical frequency, familiarity, and imageability of target stimuli were obtained from an online database^4^ (see also Wilson, 1988). Half of the categories depicted objects with high-frequency names (mean 59.72; range 41–86), whereas the other half depicted objects with low-frequency names (mean 8.75; range 5–15; see Appendix A). Lexical frequency differed significantly for high- and low-frequency categories [*t*(62) = 5.02, *p* < 0.00001], even after controlling for indices of imageability and familiarity [*F*(1, 60) = 20.44, *p* < 0.001]. Picture names were either mono- or disyllabic, and the number of syllables did not differ between high- and low-frequency categories [*t*(62) = 1.59, *p* = 0.12]. In Experiments 2 and 3, stimuli also included visually presented written word forms of the 64 target picture names.

**FIGURE 2:**
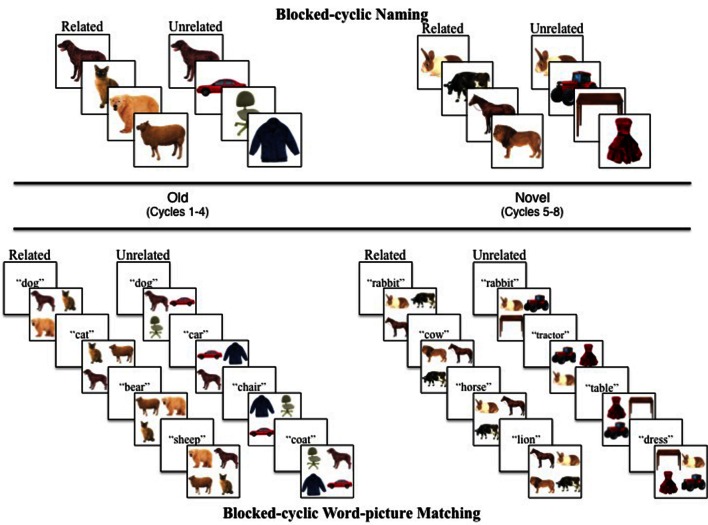
**Illustration of the blocked-cyclic naming (top panel) and word-picture matching (bottom panel) tasks.** Pictures appeared in sets of semantically related and unrelated contexts. Four items repeated (i.e., Old; **left panel)** for four cycles (i.e., Cycles 1–4) followed by the repetition of four novel items (i.e., Novel; **right panel)**. Experiment 3 followed a similar design except that participants first performed the blocked-cyclic naming (or word-picture matching) for the Old Block Half and then performed the blocked-cyclic word-picture matching (or naming) for the Novel Block Half. Subjects in Experiment 3 performed the naming task (i.e., Cycles 1–4) followed by the word-picture matching task (i.e., Cycles 5–8) for half of the blocks, and performed the tasks in the reverse order for the other half of the blocks.

Items in each of the eight semantic categories appeared together to form four high- and four low-frequency related blocks of trials consisting of eight items each. One item from each of the high- or low-frequency related categories appeared together in a set to form four high- and low-frequency unrelated blocks of trials, resulting in a total of 16 blocked sets (see Appendix B). Each block consisted of a set of four pictures that repeated for four cycles in different orders (i.e., Old) followed by four cycles repeating the remaining four pictures in the set (i.e., Novel). For example, in the Related Condition, the Old set contained four same-category pictures (e.g., animal: BEAR, CAT, LION, and SHEEP), and the Novel set contained four novel pictures drawn from the same semantic category (e.g., animal: DOG, COW, RABBIT, and HORSE). The 8-item unrelated sets contained two exemplars from each of the four high- or low-frequency semantic categories, where one appeared in the Old Block Half (e.g., BEAR, CAR, SHOE, and CHAIR) and another appeared in the Novel Block Half (e.g., DOG, VAN, SHIRT, and RUG; see **Figure 2**). Stimuli appeared an equal number of times in each condition. Blocked sets appeared in pseudorandom order, such that no more than three blocks of the same Condition (Related and Unrelated) or Frequency (High and Low) appeared consecutively. Following these constraints, we created five stimulus presentation lists. Items appearing in the Old vs. Novel sets were counterbalanced across participants to ensure that any differences between semantic interference effects in the Old and Novel Block Halves were not due to the specific items used. This resulted in a total of 10 lists of test materials. Together, there were 16 blocks with 32 trials each for a total of 512 trials per subject.

### Apparatus

Target stimuli were presented using DMDX software (Forster and Forster, 2003). To record naming performance (i.e., Experiments 1 and 3), a microphone headset triggered a voice key to collect RTs to the nearest millisecond (ms) and record verbal responses. An experimenter coded naming errors. To record word-picture matching performance (i.e., Experiments 2 and 3), participants made their response using a touch screen monitor, and DMDX software recorded RTs to the nearest ms and error data (i.e., tapping the wrong picture).

### Experiment 1: Semantic Interference in Picture Naming

To establish that semantic interference in blocked-cyclic naming originates at the semantic level but has it locus at the lexical level (e.g., Belke, 2013), we tested whether (1) repeatedly naming a set of semantically related pictures renders subsequent naming of novel pictures drawn from the same semantic category more difficult (i.e., generalization of semantic interference), and (2) whether the lexical frequency of targets affects semantic interference magnitudes. To test the prediction that interference generalizes to novel category members (Howard et al., 2006; Oppenheim et al., 2010; see also Belke et al., 2005b), in Experiment 1 we examined whether semantic interference increased when naming novel items (collapsed across cycles) in comparison to having previously named different items from the same category. Subjects named sets of semantically related and unrelated pictures across four cycles (i.e., Old) immediately followed by naming novel semantically related or unrelated pictures for an additional four cycles (i.e., Novel; following Belke et al., 2005b). While Belke et al. (2005b) found that interference emerged on the second cycle and remained stable thereafter, a computational simulation of this experiment predicts that semantic interference increases incrementally (linearly) across cycles of previously named and novel category members (Oppenheim et al., 2010, Simulation 3). In either case, larger semantic interference for novel compared to old items (collapsed across cycles) is predicted to occur because either interference is absent on the first cycle of the block and present on later cycles (Belke et al., 2005b) or because interference continues to increase linearly across cycles of both old and novel items (Oppenheim et al., 2010). Thus, although we predict increased interference for novel items categorically related to those named previously (collapsed across cycles), it remains unclear *how* semantic interference in naming generalizes to novel category members in the blocked-cyclic naming task (i.e., stable vs. linear increase), as there is limited evidence to support either account of interference generalization in this task.

To investigate the contribution of lexical-level processing on semantic interference in naming and word-picture matching, we compared semantic interference for high- vs. low-frequency picture names. The semantically related and unrelated sets consisted of objects depicting words with similar frequency counts. This was done to replicate the word-picture matching findings of Campanella and Shallice (2011), and directly compare semantic interference in word-picture matching with that of naming. If semantic interference in naming has a lexical-level locus, we predict greater interference for low- vs. high-frequency picture names because their inherently lower activation levels render them more susceptible to interference from co-activated, same-category high-frequency words with inherently higher activation levels.

#### Procedure

Prior to the experiment, subjects were familiarized with the picture stimuli and their corresponding names. In the learning phase, each picture appeared centrally on the computer screen with its written name displayed underneath the picture. The picture and name stayed on the screen until the subject pressed a key indicating that they understood the correct response for the stimulus. To keep the learning phase consistent across experiments, subjects were instructed to not name the pictures, as naming the pictures could contaminate semantic interference effects observed in the word-picture matching variants of the task (Experiments 2 and 3).

Immediately after the learning phase, the experimental phase began. A single picture appeared in the center of the screen, and subjects were instructed to name the picture as quickly and accurately as possible into the microphone headset. If the microphone failed to trigger the voice key, then the subject would see the words “Speak up” before the next picture appeared which indicated that they should speak more loudly on the next trial. The picture remained on the screen for 1600 ms or until the subject made a response (similar to previous studies using the blocked-cyclic tasks; e.g., Damian and Als, 2005; Campanella and Shallice, 2011). Once a response was made, the next trial began immediately [i.e., 0 ms response stimulus interval (RSI), following Campanella and Shallice, 2011]. Subsequent trials either depicted same category (Related Condition) or different category items (Unrelated Condition). See **Figure 2**. The experiment lasted approximately 20 min.

#### Statistical Analyses

We excluded from the analyses RTs for trials classified as an error (i.e., incorrect naming response or no response and voice-key malfunction) and responses faster than 250 ms or slower than 1550 ms (following Damian and Als, 2005). Valid RTs were analyzed using a repeated measures analysis of variance (ANOVA) with participants and items as random factors, yielding *F*_1_ and *F*_2_ statistics, respectively. Fixed factors included Condition (Related and Unrelated), Block Half (Old, Novel), Cycles (1–4), and Frequency (High-frequency and Low-frequency). All fixed factors were considered within-subject, within-item variables except for Frequency, which was a within-subject variable in the *F*_1_ analysis and a between-item variable in the *F*_2_ analysis.

### Results and Discussion

Response errors occurred on 4.4% of experimental trials. **Tables 1** and **2** summarize RT *F* statistics and mean RTs, respectively.

**Table 1:** Experiment 1 ANOVA results.

	Subject			Item		
	Degrees of freedom			Degrees of freedom		
	Numerator	Denominator	*F*_1_	Numerator	Denominator	*F*_2_
Condition	1	30	13.51*	1	62	9.89*
Block Half	1	30	33.41*	1	62	40.97*
Cycle	3	90	213.66*	3	186	195.18*
Frequency	1	30	24.07*	1	62	7.60*
Condition × Block Half	1	30	4.83*	1	62	6.10*
Condition × Cycle	3	90	11.67*	3	186	10.96*
Condition × Frequency	1	30	4.32*	1	62	2.90
Block Half × Cycle	3	90	1.90	3	186	2.39
Block Half × Frequency	1	30	0.04	1	62	0.39
Cycle × Frequency	3	90	5.65*	3	186	2.31
Condition × Block Half × Cycle	3	90	2.47	3	186	2.45
Condition × Block Half × Frequency	1	30	4.39*	1	62	4.07*
Condition × Cycle × Frequency	3	90	0.69	3	186	1.12
Block Half × Cycle × Frequency	3	90	0.99	3	186	1.45
Condition × Block Half × Cycle × Frequency	3	90	0.34	3	186	0.36

*Summary of *F* statistics for the RT ANOVA examining the effects of Condition (Related and Unrelated), Block Half (Old and Novel), Cycles (1–4), and Frequency (High-Frequency and Low-Frequency).*

Significant main effects and interactions in both the *F*_*1*_ and *F*_*2*_ analyses appear shaded in gray, and an * indicates a significant effect at *p* < 0.05.

**Table 2:** Experiment 1 naming latencies.

		Cycles							
		Old				Novel			
		1	2	3	4	5	6	7	8
Related	High-frequency	706	652	637	625	755	666	668	646
	Low-frequency	754	672	679	660	794	696	686	668
	Mean	730	662	658	642	774	681	677	657
Unrelated	High-frequency	739	658	631	614	741	667	632	619
	Low-frequency	767	648	646	617	785	674	650	639
	Mean	753	653	639	616	763	671	641	629
Difference		-22	9	19	27	12	10	35	28

*Mean RTs displayed in the shaded rows are collapsed across Frequency. Mean RTs (in ms) latencies separated by Condition, Block Half, Cycle, and Frequency.*

There were significant main effects of Condition, Block Half, Cycle, and Frequency. Participants responded more slowly in the Related (685 ms) compared to the Unrelated Condition [670 ms; Condition effect 15 ms, 95% confidence interval (CI) 7–23 ms]. RTs were faster in the Old (669 ms) vs. Novel Block Half (687 ms; Block Half effect 18 ms, 95% CI 12–24 ms). Participants also became faster across naming cycles (755, 667, 654, and 636 ms), replicating previous findings of repetition priming in studies using the blocked-cyclic naming task (e.g., Schnur et al., 2006; Navarrete et al., 2012, 2014). Lastly, naming latencies were faster for high- (666 ms) compared to low-frequency words (690 ms; Frequency effect 24 ms, 95% CI 15–33 ms), which replicates the lexical frequency effect found elsewhere (e.g., Oldfield and Wingfield, 1965; Jescheniak and Levelt, 1994; Griffin and Bock, 1998) and demonstrates the items were sensitive to this variable.^5^

**FIGURE 3:**
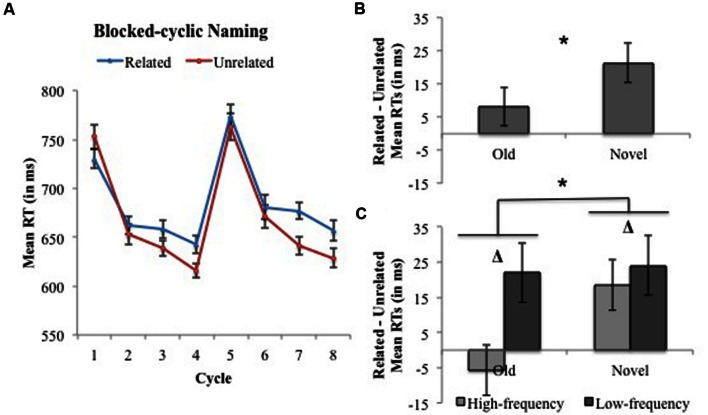
**Experiment 1: Blocked-cyclic naming. (A)** Mean response times (RTs) and associated within-subjects 95% confidence intervals (CIs) across cycles of naming semantically related (blue) and unrelated (red) pictures. Cycles 1–4 correspond to items named in the Old Block Half, whereas Cycles 5–8 correspond to items named in the Novel Block Half. **(B)** The magnitude of semantic interference (Related – Unrelated) and associated within-subjects 95% CIs collapsed across cycles separated by Old and Novel Block Halves. **(C)** The magnitude of semantic interference (Related – Unrelated) and associated within-subjects 95% CIs collapsed across cycles and separated by Old and Novel Block Halves for High- and Low-frequency categories. An * indicates a significant effect at *p* < 0.05.

Two-way interactions were significant between Condition and Block Half and Cycle. The Condition × Cycle interaction revealed that the semantic interference effect (Related – Unrelated) increased with repetition across cycles (collapsed across Block Half; -5, 9, 27, and 27 ms). The Condition × Block Half interaction revealed that semantic interference increased when naming novel (21 ms) vs. old pictures (8 ms; Condition × Block Half effect 13 ms, 95% CI 1–25 ms), indicating that semantic interference in the blocked-cyclic naming task generalizes to novel category items not previously named (Belke et al., 2005b; see **Figure 3B**). We assessed whether generalization of semantic interference manifested as a linear increase across cycles of old and novel items (Oppenheim et al., 2010, Simulation 3) vs. emerging on the second cycle and remaining stable thereafter (Belke et al., 2005b, Experiment 3). While interference increased linearly when all eight cycles are included in the analyses [*F*_1_(1,30) = 21.18, *p* < 0.001; *F*_2_(1,63) = 23.64, *p* < 0.001], the linear contrast is not significant when the first cycle is excluded from the analyses (*p*’s > 0.11), suggesting that interference emerges after Cycle 1 and remains stable thereafter (see Belke et al., 2005b). We also conducted analyses including Cycles 2–5 following the prediction put forth in Belke et al. (2005b, p. 683): “If the semantic blocking effect generalizes to new items, the difference between homogeneous and heterogeneous sets that we expected to observe in cycles 2–4 should prevail on the 5th cycle.” Consistent with Belke et al. (2005b) we find a significant main effect of Condition [*F*_1_(1,30) = 12.15, *p* = 0.002; *F*_2_(1,63) = 11.43, *p* = 0.001] and Cycle [*F*_1_(3,90) = 222.35, *p* < 0.001; *F*_2_(3,189) = 199.10, *p* < 0.001], but no interaction between the two variables (*F*’s < 1.73, *p*’s > 0.16). Consistent with generalization of interference as defined in Forde and Humphreys (1995, Experiment 12), we also find larger semantic interference on the first cycle of novel items (i.e., Cycle 5) vs. the first cycle of old items (i.e., Cycle 1) [Related – Unrelated 12 ms vs. -22 ms, respectively; *F*_1_(1,30) = 16.01, *p* = 0.002; *F*_2_(1,63) = 12.74, *p* = 0.001].

Analyses examining Frequency revealed marginally significant interactions between this variable and Cycle and Condition – but a significant three-way interaction between Frequency, Condition, and Block Half. The Frequency × Cycle marginal interaction was due to a reduction in the lexical frequency effect (High- < Low-frequency) with repetition (Low-frequency – High-frequency difference: 39, 12, 23, and 20 ms), which is consistent with previous studies (e.g., Scarborough et al., 1977; Jescheniak and Levelt, 1994; Griffin and Bock, 1998). The marginal interaction between Frequency and Condition indicated that low- compared to high-frequency items exhibited greater semantic interference (Related – Unrelated; 23 vs. 6 ms, respectively; Condition × Frequency effect 17 ms, 95% CI 1–32 ms). The Frequency × Cycle and Frequency × Condition interactions were significant by subject and marginally significant by item (Frequency × Cycle: *p* = 0.07; Frequency × Condition: *p* = 0.09), which may be because Frequency is a between-item variable in the *F*_2_ analyses, and the significant three-way interaction between Frequency, Condition, and Block Half. That is, semantic interference for low-frequency words exceeded that of high-frequency words in the Old Block Half [22 ms vs. -6 ms, respectively; *F*_1_(1,30) = 7.92, *p* < 0.001; *F*_2_(1,62) = 7.53, *p* < 0.001], but did not differ from high-frequency words in the Novel Block Half [24 ms vs. 19 ms, respectively; *F*_1_(1,30) = 0.35, *p* = 0.56; *F*_2_(1,62) = 0.23, *p* = 0.63; see **Figure 3C**).^6^

To summarize, the results from Experiment 1 are consistent with the assumption that semantic interference in naming originates at the semantic level but has its locus at the lexical level (e.g., Belke, 2013). That semantic interference increased when naming novel items categorically related to those named previously (collapsed across cycles) demonstrates that interference originates at the semantic level as a result of shared activation. However, inconsistent with computational models of semantic interference in naming (e.g., Oppenheim et al., 2010; see also Howard et al., 2006) is the finding that the effect does *not* increase linearly across cycles of old and novel items. Instead, we replicate evidence elsewhere demonstrating that interference emerges on the second cycle and remains stable thereafter (Belke et al., 2005b), resulting in larger interference for the first cycle of novel vs. the first cycle of old items (Forde and Humphreys, 1995). This suggests that the larger interference effect observed for Novel relative to Old Block Halves (collapsed across cycles) reflects in part the presence of facilitation on the first cycle of old items vs. its absence on the first cycle of the novel items.

In addition, lexical frequency modulated semantic interference in naming, providing evidence of a lexical-level locus. However, the three-way interaction between lexical frequency, semantic interference, and Block Half (Old vs. Novel) was surprising, as it might be expected that lexical frequency would have a consistent impact on interference across both old and novel items. We discuss this unexpected finding in the General Discussion, as it has implications for different mechanistic accounts of *how* semantic interference in naming occurs, i.e., due to competitive lexical selection (e.g., Roelofs, 1992; Levelt et al., 1999; Howard et al., 2006) vs. competitive learning (Oppenheim et al., 2010). Nonetheless, the results from Experiment 1 support a semantic origin and lexical locus of the interference effect in naming.

### Experiment 2: Semantic Interference in Word-Picture Matching

Although the locus of interference is thought to differ in the two tasks – lexical in naming (e.g., Howard et al., 2006; Oppenheim et al., 2010) and semantic in word-picture matching (e.g., Forde and Humphreys, 1997; Campanella and Shallice, 2011) – interference is assumed to originate at a shared semantic level. Thus, interference in word-picture matching is predicted to generalize to novel category items. Moreover, qualitative RT patterns suggest that semantic interference increases for low- relative to high-frequency words in word-picture matching (Campanella and Shallice, 2011), raising the possibility that interference in naming and word-picture matching have a shared origin *and* locus. We test this hypothesis in Experiment 2 using a word-picture matching variant of the blocked-cyclic naming task used in Experiment 1. In this task, subjects matched a visually presented word to its corresponding picture which appeared embedded in an array of three distractor pictures either semantically related or unrelated to the target picture (e.g., Biegler et al., 2008, Experiment 2B; Harvey and Schnur, 2015). If the locus of interference is shared across the two tasks, then semantic interference in word-picture matching will exhibit the same characteristics as those observed in picture naming (Experiment 1): increased semantic interference for novel compared to old categorically related words (i.e., generalization of semantic interference) and greater interference for low- compared to high-frequency words for old vs. novel pictures (i.e., an interaction between Condition, Frequency, and Block Half). Alternatively, if semantic interference in word-picture matching has its origin *and* locus within the semantic system (e.g., Forde and Humphreys, 1997, 2007; Campanella and Shallice, 2011), then it is predicted to generalize to novel category members, but not interact with lexical frequency.

#### Procedure

Subjects first completed the learning phase identical to that of Experiment 1. Immediately thereafter, subjects completed a practice phase, which followed the same parameters as the actual experiment but used items not depicted in the experiment (i.e., Old: BEE, ORANGE, SCISSORS, and DOLFIN; Novel: PLANT, GRAPES, PEN, and SHARK). This was done to demonstrate the speed with which the target words appear in the blocked-cyclic word-picture matching task. The procedure was as follows. A visually presented word appeared in the center of the screen for 300 ms followed by an array of four pictures: one corresponding to the previous target word and three distractor pictures. Subjects were instructed to select the picture that matches the previously presented word by tapping the picture on the touch screen monitor. Distractor pictures depicted words appearing as other targets in the cycle, and therefore either belonged to the same semantic category as the target (Related Condition) or belonged to different semantic categories as the target (Unrelated Condition). See **Figure 2**. All other experiment parameters were identical to Experiment 1.

#### Statistical Analyses

Response times analyses did not include trials classified as an error (i.e., selecting an incorrect picture) or responses faster than 250 ms or slower than 1550 ms. Valid RTs were analyzed using the same repeated measures ANOVAs as those used in Experiment 1: Random factors included participants and items, yielding *F*_1_ and *F*_2_ statistics, respectively. Fixed factors included Condition (Related and Unrelated), Block Half (Old and Novel), Cycles (1–4), and Frequency (High-frequency and Low-frequency). All fixed factors were considered within-subject, within-item variables except for Frequency, which was a within-subject variable in the *F*_1_ analysis and a between-item variable in the *F*_2_ analysis.

### Results and Discussion

Response errors occurred on 1.1% of experimental trials. **Tables 3** and **4** summarize RT *F* statistics and mean RTs, respectively. See **Figure 3A** for the full pattern of results.

**Table 3:** Experiment 2 ANOVA results.

	Subject			Item		
	Degrees of freedom			Degrees of freedom		
	Numerator	Denominator	*F*_1_	Numerator	Denominator	*F*_2_
Condition	1	19	97.42*	1	62	29.71*
Block Half	1	19	1.35	1	62	0.10
Cycle	3	57	7.81*	3	186	1.41
Frequency	1	19	0.05	1	62	0.12
Condition × Block Half	1	19	1.99	1	62	0.03
Condition × Cycle	3	57	1.24	3	186	0.64
Condition × Frequency	1	19	1.91	1	62	4.42*
Block Half × Cycle	3	57	1.12	3	186	1.14
Block Half × Frequency	1	19	0.43	1	62	0.38
Cycle × Frequency	3	57	2.21	3	186	0.95
Condition × Block Half × Cycle	3	57	0.38	3	186	1.57
Condition × Block Half × Frequency	1	19	0.05	1	62	0.06
Condition × Cycle × Frequency	3	57	0.75	3	186	1.28
Block Half × Cycle × Frequency	3	57	2.65	3	186	1.33
Condition × Block Half × Cycle × Frequency	3	57	1.20	3	186	0.72

Significant main effects and interactions in both the *F*_*1*_ and *F*_*2*_ analyses appear shaded in gray, and an * indicates a significant effect at *p* < 0.05. Summary of *F* statistics for the RT ANOVA examining the effects of Condition (Related and Unrelated), Block Half (Old and Novel), Cycles (1–4), and Frequency (High-frequency and Low-frequency).

**Table 4:** Experiment 2 word-picture matching response latencies.

		Cycles							
		Old				Novel			
		1	2	3	4	5	6	7	8
Related	High-frequency	702	671	661	663	701	658	669	703
	Low-frequency	687	658	668	659	694	694	665	663
	Mean	695	664	665	661	698	676	667	683
Unrelated	High-frequency	618	587	585	592	608	577	584	597
	Low-frequency	599	590	617	593	621	589	595	605
	Mean	609	589	601	593	614	583	590	601
Difference	86	75	63	68	83	93	77	82

*Mean RTs displayed in the shaded rows are collapsed across Frequency. Mean RTs (in ms) separated by Condition, Block Half, Cycle, and Frequency.*

**FIGURE 4:**
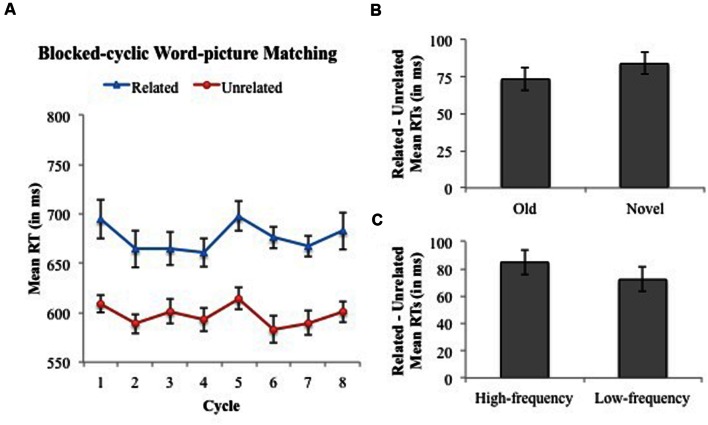
**Experiment 2: Blocked-cyclic word-picture matching. (A)** Mean RTs and associated within-subjects 95% CIs across cycles of matching words to pictures in the related (blue) vs. unrelated (red) condition. Cycles 1–4 correspond to items that appeared in the Old Block Half, whereas Cycles 5–8 correspond to items that appeared in the Novel Block Half. **(B)** The magnitude of semantic interference (Related – Unrelated) and associated within-subjects 95% CIs collapsed across cycles separated by Old and Novel Block Halves. **(C)** The magnitude of semantic interference (Related – Unrelated) and associated within-subjects 95% CIs collapsed across cycles and separated by High- and Low-frequency categories.

There was a main effect of Condition, due to slower RTs in the Related (676 ms) compared to the Unrelated Condition (598 ms; Condition effect 79 ms, 95% CI 63–94 ms). However, in contrast to the naming results obtained in Experiment 1, main effects of Block Half, Frequency, and Cycle were not significant. Lastly, two- and three-way interactions between Condition, Block Half, and Frequency were not significant (see **Figure 4**). As in Experiment 1, we examined interference generalization in word-picture matching following Belke et al. (2005b; i.e., stable interference across Cycles 2–5) and Forde and Humphreys (1995; i.e., larger Cycle 5 vs. Cycle 1 interference). We found that although interference remained stable across Cycles 2–5 (i.e., main effect of Condition [*F*_1_(1,19) = 74.62, *p* < 0.001; *F*_2_(1,63) = 33.17, *p* < 0.001], but no interaction with Cycle (*F*’s < 1.19, *p*’s > 0.31)), interference did *not* differ on the first cycle of old vs. the first cycle of novel items (Related – Unrelated 86 ms vs. 83 ms, respectively; *F*’s < 0.07, *p*’s > 0.80). Together, these findings demonstrate that although semantic interference occurs in healthy participants’ word-picture matching performance, it does *not* manifest in the same manner as that which occurs in picture naming (i.e., Experiment 1).

The findings from Experiment 2 are only partially consistent with the assumption that the origin and locus of semantic interference in word-picture matching is at the semantic level (e.g., Warrington and Cipolotti, 1996; Forde and Humphreys, 2007) for two reasons. First, interference did not increase for novel relative to old category items (i.e., no generalization of interference) – a result at odds with a previous neuropsychological finding suggesting a semantic-level locus (Forde and Humphreys, 1995, Experiment 12). It is possible that the visual similarity between target and distractor pictures in related vs. unrelated picture arrays contaminates the interference effect, masking potential changes in interference across cycles. Belke et al. (2005b) suggested that in naming, the absence of interference on the first cycle argues against a visual (similarity) locus of the effect, as interference due to visual similarity should be largest at the first presentation of items (i.e., Cycle 1) and reduced on subsequent presentations. We had participants from Experiment 2 rate the visual similarity of target pictures appearing together in an array after completing the word-picture matching task to assess the contribution of visual similarity on word-picture matching RTs. Two pictures appeared side-by-side, and participants rated the visually similarity of the two pictures on a 5-point scale (1 = not at all visually similar, 5 = very highly visually similar). Items appearing in the related vs. unrelated picture arrays were rated as more visually similar [*t*_1_(19) = 9.46, *p* < 0.001; *t*_2_(63) = 14.41, *p* < 0.001]. An analysis of covariance revealed that the condition effect remained significant after controlling for visual similarity in the analysis by subject [*F*_1_(1,18) = 15.35, *p* = 0.001], but not by item [*F*_2_(1,62) = 2.20, *p* = 0.14], suggesting that for some of the picture arrays visual similarity contributed to the interference effect.

Second, consistent with a non-lexical locus of interference (e.g., Forde and Humphreys, 1997, 2007), interference was *not* sensitive to lexical frequency, a hypothesized lexical, not semantic effect. This is in contrast to Campanella and Shallice (2011) who found that lexical frequency had some effect on semantic interference in word-picture matching. While it is not entirely clear why we did not replicate Campanella and Shallice, we hypothesize it may be due to a difference between experiment designs. Campanella and Shallice (Experiment 1) included a baseline (unrelated) condition but the items in this baseline condition differed from those tested in the experimental (related) condition (and cycle was not manipulated as a factor). In contrast, in the present study, items served as their own controls by appearing in both the related and unrelated conditions (allowing us to draw stronger conclusions concerning the effects of relatedness across items; see also Biegler et al., 2008; Harvey and Schnur, 2015; Wei and Schnur, 2016). Thus, it may be the case that their findings were driven by item-specific differences between related and unrelated conditions.

### Experiment 3: Transfer of Semantic Interference

Experiments 1 and 2 demonstrated that the locus of interference in naming and word-picture matching tasks differs, but leave open the possibility that semantic interference originates at a shared processing level. Experiment 3 tested this hypothesis by investigating whether accessing the semantic system when performing the word-picture matching task with semantically related vs. unrelated words subsequently impacts naming novel pictures drawn from the same vs. different semantic categories.^7^ If the origin of semantic interference in the two tasks is *not* shared, then semantic interference when naming before (i.e., Old) and after word-picture matching (i.e., Novel) will be of comparable magnitudes, i.e., unaffected by interference in word-picture matching. However, if interference in word-picture matching and naming originate at a shared level, then semantic interference should transfer across tasks, whereby naming novel pictures semantically related to those accessed previously in word-picture matching should result in greater semantic interference than when naming precedes word-picture matching (i.e., novel > old same-category naming).

#### Procedure

The procedure followed that of the previous experiments except in Experiment 3 subjects either named pictures or performed word-picture matching in the first Block Half (i.e., Old) and switched tasks for the second Block Half (i.e., Novel) within both related and unrelated blocks of trials (see **Figure 2**). For a given subject, half of the blocks began with word-picture matching (i.e., Cycles 1–4) followed by naming (i.e., Cycles 5–8), whereas the other half began with naming (i.e., Cycles 1–4) followed by word-picture matching (i.e., Cycles 5–8). While we did not expect to find changes when switching from naming to word-picture matching (see footnote 6), we included this manipulation to determine if the semantic interference magnitude for naming increased in the Novel Block Half (i.e., after word-picture matching) relative to naming in the Old Block Half (i.e., before word-picture matching), where a larger magnitude when switching to naming would indicate interference transferred from word-picture matching to picture naming. Subjects always switched tasks halfway through the block (i.e., when presented with cycles of novel stimuli) an equal number of times throughout the experiment. The order of the task switch (from naming to word-picture matching and vice versa) was constrained so that no more than three consecutive blocks occurred with the same task switching direction. Further, there were an equal number of Related and Unrelated as well as High- and Low-frequency blocks for each task switching direction. We created an additional 10 lists of test materials by counterbalancing across subjects items appearing in each Block Half for a given task (i.e., blocked-cyclic naming or word-picture matching), resulting in a total of 20 lists.

As in Experiment 2, participants completed a practice phase with the same stimuli used in Experiment 2 to familiarize them with not only the fast presentation rate, but also the task-switching procedure. Because the task-switching was somewhat unpredictable, subjects were given a cue (i.e., #####) when the task switched from word-picture matching to naming, and the visually presented target word served as a cue when switching from naming to word-picture matching. Thus, participants always practiced the word-picture matching task before the picture naming task in order to familiarize them with the switch cue. When a single picture appeared on the screen, subjects were instructed to name the picture into the microphone headset as quickly and accurately as possible. When a word appeared followed by an array of pictures, subjects were instructed to select the picture corresponding to the previously presented word by tapping the picture on the touch screen monitor. All other experiment parameters were identical to Experiments 1 and 2.

#### Statistical Analyses

Response times for trials were excluded in the same manner as Experiments 1 and 2. Valid RTs were analyzed using repeated measures ANOVAs with participants and items as random factors, yielding *F*_1_ and *F*_2_ statistics, respectively. Fixed factors included: Task (Blocked-cyclic naming and Blocked-cyclic word-picture matching), Condition (Related and Unrelated), Block Half (Old vs. Novel), and Cycles (1–4). All fixed factors were considered within-subject, within-item variables.

### Results and Discussion

Response errors occurred on 3.9% of experimental trials. **Tables 5** and **6** summarize RT *F* statistics and mean RTs, respectively.

**Table 5:** Experiment 3 ANOVA results.

	Subject			Item		
	Degrees of freedom			Degrees of freedom		
	Numerator	Denominator	*F*_1_	Numerator	Denominator	*F*_2_
Task	1	42	9.83*	1	63	37.77*
Condition	1	42	249.21*	1	63	73.30*
Block Half	1	42	0.01	1	63	0.09
Cycle	3	126	155.33*	3	189	197.44*
Task × Condition	1	42	152.87*	1	63	80.41*
Task × Block Half	1	42	1.31	1	63	2.28
Task × Cycle	3	126	26.88*	3	189	23.31*
Condition × Block Half	1	42	0.00	1	63	0.09
Condition × Cycle	3	126	1.49	3	189	1.47
Block Half × Cycle	3	126	21.25*	3	189	23.64*
Task × Condition × Block Half	1	42	9.01*	1	63	10.76*
Task × Condition × Cycle	3	126	2.24	3	189	1.77
Task × Block Half × Cycle	3	126	2.95*	3	189	1.88
Condition × Block Half × Cycle	3	126	1.89	3	189	2.85*
Task × Condition × Block Half × Cycle	3	126	0.87	3	189	1.23

Significant main effects and interactions in both the *F*_*1*_ and *F*_*2*_ analyses appear shaded in gray, and an * indicates a significant effect at *p* < 0.05. Summary of *F* statistics for the RT ANOVA examining the effects of Task (Blocked-cyclic naming and Blocked-cyclic word-picture matching), Condition (Related and Unrelated), Block Half (Old and Novel), and Cycles (1–4).

**Table 6:** Experiment 3 response latencies.

		Cycles							
		Old				Novel			
		1	2	3	4	5	6	7	8
Blocked-cyclic naming	Related	731	647	652	641	763	648	638	640
	Unrelated	751	655	644	627	752	631	618	608
	Difference	-20	-8	8	14	11	17	20	32
	


	Mean	741	651	648	634	758	640	628	624
Blocked-cyclic Word-picture matching	Related	784	750	743	751	822	728	712	750
	Unrelated	673	651	632	647	733	636	652	651
	Difference	111	99	111	104	90	93	60	100
	


	Mean	729	701	687	699	778	682	682	700

*Mean RTs displayed in the shaded rows are collapsed across Condition. Mean RTs reflect data from all participants, as each participant performed the naming and word-picture matching tasks in both the Old and Novel Block Halves (see Procedure in Experiment 3). Mean RTs (in ms) separated by Task, Condition, Block Half, and Cycle.*

**FIGURE 5:**
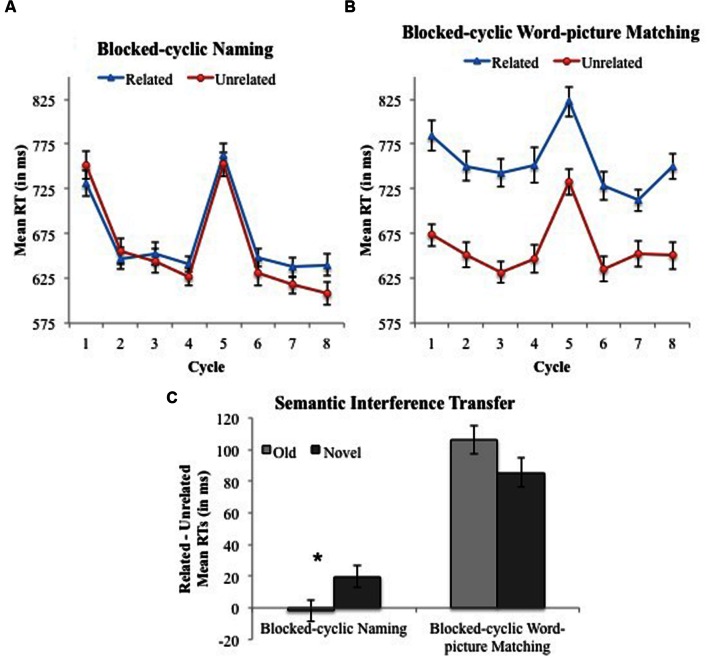
**Experimen 3: Blocked-cyclic naming and word-picture matching.** Mean RTs and associated within-subjects 95% CIs across cycles of naming pictures and matching words to pictures in the semantically related (blue) and unrelated (red) conditions. **(A)** Cycles 1–4 correspond to items named in the Old Block Half prior to performing the word-picture matching task, whereas Cycles 5–8 correspond to items named in the Novel Block Half after having performed the word-picture matching task with semantically related words in the Old Block Half. **(B)** Cycles 1–4 correspond to items that appeared in the word-picture matching task in the Old Block Half prior to naming pictures, whereas Cycles 5–8 correspond to items that appeared in the word-picture matching task in the Novel Block Half after having named semantically related pictures in the Old Block Half. **(C)** The magnitude of semantic interference (Related – Unrelated) and associated within-subjects 95% CIs collapsed across cycles and separated by Old and Novel Block Halves for blocked-cyclic naming and word-picture matching. Here, “Old” bars illustrate the semantic interference effect observed before switching tasks, whereas “Novel” refers to the semantic interference effect observed after switching tasks. An * indicates a significant effect at *p* < 0.05.

There were main effects of Task, Condition, and Cycle. The effect of Task revealed that RTs were faster for blocked-cyclic naming (666 ms) compared to word-picture matching (707 ms; Task effect 41 ms, 95% CI 15–67 ms). As expected, the main effect of Condition was due to slower RTs in the Related (713 ms) vs. Unrelated Condition (660 ms; Condition effect 53 ms, 95% CI 47–60 ms). The Cycle effect was due to decreasing response latencies from the first (751 ms) to the remaining three cycles (669, 662, and 665 ms; see **Figures 5A,B**).

The interaction between Task and Cycle revealed that RTs decreased with repetition to a greater extent in blocked-cyclic naming (749, 645, 638, and 629 ms) than word-picture matching (753, 691, 684, and 700 ms), and Block Half also interacted with Cycle, but not in a meaningful way. The interaction between Task and Condition was due to smaller semantic interference (Related – Unrelated) in naming (9 ms) than word-picture matching (96 ms; Task × Condition difference 87 ms, 95% CI 73–101 ms). The significant three-way interaction between Task, Condition, and Block Half was due to larger semantic interference when naming in the Novel Block Half (i.e., after word-picture matching) compared to naming in the Old Block Half (20 ms vs. -2 ms, respectively). The increase in naming-induced semantic interference for Novel vs. Old Block Halves was confirmed with a simple effects comparison [Condition × Block Half effect 22 ms, 95% CI 7–35 ms; *t*_1_(42) = -3.01, *p* < 0.01; *t*_2_(63) = -2.69, *p* < 0.01]. That interference in naming was greater following word-picture matching suggests that semantic interference transferred from word-picture matching to picture naming (see **Figure 5C**).

We were surprised, however, to find that interference in the Old Block Half of naming was numerically smaller in Experiment 3 (-2 ms) compared to Experiment 1 (8 ms). We assessed *post hoc* whether interference in naming across the two experiments was similar in magnitude (thus similar in terms of “generalization”) with repeated measures ANOVAs that included Experiment (Experiments 1 and 3) as a between-subjects, within-item variable. The results mirrored the main findings of interference in naming reported above (i.e., main effects of Condition, Related > Unrelated), Cycle (RTs decreased across cycles), and interactions between Condition and Block Half (Novel > Old) and Condition and Cycle (semantic interference increased across cycles; *F*’s > 10.35, *p*’s < 0.002). Critically, the factor Experiment did not modulate any interactions with Condition (*F*’s < 1.45, *p*’s > 0.23). The increase in semantic interference from old to novel same-category naming was the same regardless of the task performed in the Old Block Half (i.e., 21 ms after naming in Experiment 1 and 20 ms after word-picture matching in Experiment 3). Likewise, analyses of interference across Cycles 2–5 (i.e., Belke et al., 2005b) and comparisons between interference on Cycle 1 vs. 5 (Forde and Humphreys, 1995) were consistent with the findings of Experiment 1 [i.e., main effects of Condition and Cycle (*F*’s > 9.75, *p*’s < 0.005), but no Condition × Cycle interaction (*F*’s < 2.17, *p*’s > 0.09), and greater Cycle 5 vs. Cycle 1 interference (Related – Unrelated = 12 ms vs. -21 ms, respectively; *F*’s > 12.95, *p*’s < 0.001)], where here too Experiment did not modulate interactions with Condition (*F*’s < 1.90, *p*’s > 0.17). This suggests that interference in naming and word-picture matching originate at a shared (semantic) level of the language system (see Belke, 2013 for a similar rationale).

## General Discussion

To bridge the gap between theories of lexical-semantic access in naming vs. word-picture matching tasks (e.g., Forde and Humphreys, 1997, 2007; Levelt et al., 1999), we examined whether the origin and/or locus of semantic interference is shared across the two tasks. Accordingly, we tested the extent to which interference generalized to novel category items and interacted with lexical frequency in picture naming and word-picture matching variants of the blocked-cyclic paradigm, while also assessing whether interference transferred across the two tasks. In line with a semantically mediated lexical locus of interference in naming (cf. Belke, 2013), Experiment 1 demonstrated that semantic interference increased when naming novel pictures drawn from the same category as those named previously where the effect differed based on the lexical frequency of target items. Experiment 2 demonstrated that although interference occurs in word-picture matching, it did not change in magnitude for old vs. novel items or for high- vs. low-frequency words. Lastly, Experiment 3 revealed that semantic interference increased when naming novel items categorically related to those accessed previously in word-picture matching (as compared to when naming preceded word-picture matching). Together, these experiments suggest that the locus of semantic interference in picture naming and word-picture matching differs (lexical vs. semantic), but that both interference effects originate at a shared (semantic) level. In the following, we discuss how these findings inform existing theories of lexical-semantic access and semantic interference phenomena in the blocked-cyclic paradigms.

### Semantic Interference in Naming

That semantic interference in naming generalized to novel category pictures and differed based on lexical frequency is consistent with a semantically mediated lexical locus of the effect. However, in order to account for the full pattern of results, additional assumptions must be adopted. For example, Oppenheim et al. (2010) predicts linearly increasing semantic interference across cycles of old and novel items (Simulation 3). However, this prediction was not confirmed, as semantic interference remains stable from when it emerged on the second cycle to the introduction of novel category items (seen here in Experiment 1 and in Belke et al., 2005b, Experiment 3). Although there may be increasing semantic interference across cycles in the blocked-cyclic naming task, according to Belke (2008) and Belke and Stielow (2013), this task promotes the use of top-down cognitive control processes which masks the accumulation of semantic interference across cycles by biasing activation toward within-set category representations and away from set-external category members (i.e., biased selection account; see also Thompson-Schill and Botvinick, 2006; Belke, 2013; Crowther and Martin, 2014). Thus, top-down control may reduce, if not eliminate, semantic interference on the first cycle of novel items (i.e., Cycle 5), providing an explanation for why the Oppenheim et al. (2010) Simulation 3 does not fully capture the lack of interference change across cycles.

Although we find that interference in naming interacted with lexical frequency – consistent with a lexical-level locus (Roelofs, 1992; Levelt et al., 1999; Howard et al., 2006; Oppenheim et al., 2010), the finding that lexical frequency differentially impacted interference for old vs. novel items was not expected. Models that assume interference occurs due to competitive lexical selection (e.g., Roelofs, 1992; Levelt et al., 1999; Howard et al., 2006) predict greater interference for both old and novel low-frequency words due to their inherently lower activation levels, which makes them more susceptible to competition from related high-frequency words. However, a recent account proposes that interference arises due to a learning mechanism that strengthens target lexical-semantic connections while weakening those of related representations (i.e., competitive learning; Oppenheim et al., 2010). Lexical-semantic connections change in magnitude (strengthen or weaken) in proportion to their activation levels, or error in becoming active on a given trial (i.e., delta rule learning; e.g., Chang et al., 2000; Gupta and Cohen, 2002). Naming the same low- vs. high-frequency words (i.e., old items) results in greater semantic interference because low-frequency words have a greater learning potential due to their inherently lower activation levels (e.g., Oldfield and Wingfield, 1965; Morton, 1969). However, novel high-frequency words will be more active than novel low-frequency words due to their inherently higher activation levels, and thus greater “unlearning” potential. In turn, novel high- compared to low-frequency related words should undergo greater lexical-semantic connection weight weakening, rendering them functionally similar to low-frequency words. Thus, the competitive learning account provides a potential explanation as to why the interaction between frequency and semantic interference differed for old vs. novel items. Although this account can only be verified by computational modeling, on the face of it, the competitive learning account explains both the finding of increased interference for low-frequency words and the interaction between this characteristic and naming old vs. novel items. However, this extension of Oppenheim et al.’s (2010) account would also predict repetition (across cycles) modulates the observed interactions between lexical frequency and semantic interference – a prediction that was not borne out by the results. That both generalization of semantic interference and lexical frequency were not sensitive to repetition (i.e., cycle) suggests that there may be other factors at play in the blocked-cyclic naming task. Future work is needed to clarify the different mechanisms underlying semantic interference when repeatedly naming the same and novel categorically related high- vs. low-frequency words in the blocked-cyclic naming task.

### Semantic Interference in Word-Picture Matching

Semantic interference in the word-picture matching task differed from that observed in picture naming, suggesting that the locus of interference differs from naming. Although we observed semantic interference in word-picture matching (replicating previous results, e.g., Biegler et al., 2008; Campanella and Shallice, 2011), interference did not increase from old to novel categorically related words, which is inconsistent with a semantic-level origin and/or locus of the effect. However, there are several other explanations to consider. First, as discussed in Experiment 2, because interference did not change across cycles but was of the same magnitude from the first cycle onward, this makes it impossible to detect any generalization (or change) from old to novel items. Other variants of the word-picture matching task may be more sensitive to change of interference and thus may provide better tools to investigate generalization (cf. Wei and Schnur, 2016). Our findings suggest that the high degree of visual similarity among related vs. unrelated picture arrays had some effect on the magnitude of interference in word-picture matching (see Results and Discussion in Experiment 2). However, consistent with a semantic-, and not lexical-level locus of interference (e.g., Warrington and Cipolotti, 1996; Gotts and Plaut, 2002; Forde and Humphreys, 2007), lexical frequency did not affect semantic interference magnitudes in word-picture matching. On the assumption that lexical frequency reflects word-level processing, this factor should *only* affect the time it takes to recognize the target word (e.g., Scarborough et al., 1977), and should *not* affect the time it takes to select the word’s depicted referent. This is because the word is presented first for a fixed amount of time, and differences in RTs for each condition reflect differences in the time it takes to select the picture rather than recognizing the word itself. Nonetheless, the failure to detect lexical frequency semantic interference effects in the word-picture matching task is consistent with a semantic-level locus of interference, but future work is needed to determine if a semantic locus of interference allows for generalization to novel word meanings using a task that minimizes potential confounds such as visual similarity among distractors pictures in the related vs. unrelated arrays.

### Transfer of Semantic Interference across Tasks

Lastly, the transfer of semantic interference from word-picture matching to naming suggests overlap in where interference originates in the two tasks. Because the naming results suggest a semantic origin and lexical locus of interference, whereas the word-picture matching results suggest a non-lexical locus of interference, together this suggests that the common origin of interference across the tasks is a semantic one. However, what is the evidence to rule out a lexical locus? First, lexical frequency did not interact with semantic interference in word-picture matching suggesting a non-lexical locus (Experiment 2). Second, although participants may have tacitly named the targets and pictures in the array (cf. Biegler et al., 2008), allowing semantic interference to seemingly transfer across tasks, there is evidence which argues against this possibility. For example, it is unlikely that subjects covertly named the four pictures in the array before responding because in Experiment 3, subjects named one picture on average within 667 ms, whereas average RTs for the word-picture matching task were 708 ms. Additionally, if subjects named the targets during the word-picture matching task, then semantic interference should have manifested as it did for naming (i.e., interacted with block half and lexical frequency). Previous work also demonstrates that semantic interference occurs in word-picture matching tasks that do *not* promote a silent naming strategy (by requiring subjects to select the picture most associated with the word), regardless of whether the distractor pictures in the array are related (Biegler et al., 2008, Experiment 3) or unrelated to the target picture (Wei and Schnur, 2016). That semantic interference occurs when matching associatively related words and pictures but does *not* occur when naming associatively related pictures (unless participants are primed with the scene/event name characterizing the associative relationship; Abdel Rahman and Melinger, 2011; cf. de Zubicaray et al., 2014), suggests that interference in word-picture matching is not due to silent naming. Third, models of semantic interference in naming (Howard et al., 2006; Oppenheim et al., 2010) assume that interference occurs *only* after having previously named from the category, and therefore do *not* predict that accessing the semantic system in word-picture matching leads to interference when subsequently naming novel category pictures. Thus, the transfer of semantic interference from word-picture matching to naming indicates a shared semantic-level origin of the effect. However, because interference did not change in magnitude when word-picture matching was tested alone (Experiment 2), we were unable to examine interference transfer from naming to word-picture matching. Consequently, future work is needed to better understand the mechanisms by which semantic interference arises in word-picture matching and those that allow for interference to transfer across tasks.

## Conclusion

We examined whether the origin and/or locus of semantic interference in picture naming and word-picture matching is shared. We found that interference in naming generalized to novel category members and interacted with lexical frequency, a pattern that supports a semantic origin and lexical locus of interference in naming. In word-picture matching, however, the evidence for a semantic-level origin and locus of interference was mixed. We observed semantic interference which did not interact with lexical frequency, suggesting a non-lexical locus of the effect. Yet interference did not generalize to novel category members, arguing against a semantic locus of the effect. Because semantic interference in word-picture matching transferred to picture naming, this suggests a common origin of interference in the two tasks. We propose the shared origin is at the semantic level, whereby accessing semantic representations contributes to interference effects in both tasks. However, future research is needed to provide more conclusive evidence regarding the shared origin of semantic interference and the mechanism by which interference transfers across word-picture matching and naming tasks.

## Author Contributions

All authors listed, have made substantial, direct and intellectual contribution to the work, and approved it for publication.

## Conflict of Interest Statement

The authors declare that the research was conducted in the absence of any commercial or financial relationships that could be construed as a potential conflict of interest.

## Footnotes

1 We use quotations to denote lexical representations and picture name responses (e.g., “dog”), whereas capitalization denotes the semantic representation corresponding to a word (e.g., DOG).

2 Damian et al. (2001) do not report if the facilitation observed in the orientation task was statistically significant. However, the RTs reported for 10 subjects are in the predicted direction: orientation judgments were faster for semantically related compared to unrelated objects (i.e., 388 ms vs. 396 ms, respectively).

3 Most agree that lexical frequency does *not* reflect semantic-level processes (e.g., Wingfield, 1968; Bartram, 1976; Meyer et al., 1998), but whether frequency has a lexical or phonological locus is debated (e.g., Jescheniak and Levelt, 1994; Finocchiaro and Caramazza, 2006; Navarrete et al., 2006). Because practice via frequently producing a word most likely strengthens connections between semantic-to-lexical *and* lexical-to-phonological representational levels, it is likely that lexical frequency effects are represented at both levels (if two levels are assumed: lexical vs. phonological; e.g., cf. Caramazza, 1997; Levelt et al., 1999).

4 http://websites.psychology.uwa.edu.au/school/MRCDatabase/uwa_mrc.htmabase/uwa_mrc.htm

5 Although the effects of lexical frequency described here could be due to either frequency or age of acquisition (they are highly correlated due to the fact that frequently produced words tend to be those acquired early in life e.g., Brysbaert and Ghyselinck, 2006, also see Barry et al., 2001), both are thought to reflect processes occurring when mapping meanings to words for naming (e.g., Belke et al., 2005a; Anderson, 2008).

6 Interference for low- but not high-frequency words decreased numerically from the last cycle of old to the first cycle of novel items (low frequency Cycle 4 vs. Cycle 5 interference effect = 43 ms vs. 9 ms; high-frequency Cycle 4 vs. Cycle 5 interference effect = 11 ms vs. 14 ms; see **Table 2**), suggesting that lexical frequency differentially affects interference magnitudes across cycles, and thus interference generalization. However, *post hoc* analyses examining whether the interference effect across Cycles 1–8 and 2–5 differed for high- vs. low-frequency words were *not* significant (*F*’s < 1.30, *p*’s > 0.25 and *F*’s < 1.68, *p*’s > 0.17, respectively).

7 Because we found that semantic interference remained stable across old and novel items in word-picture matching, it would not be expected to increase for novel items semantically related to those tested in a previous block or task. Thus, the failure to demonstrate interference generalization in word-picture matching precludes inferences concerning interference transfer from naming to word-picture matching.
