# Supplementary material for: Different Loci of Semantic Interference in Picture Naming vs. Word-Picture Matching Tasks
Source: Front Psychol. 2016 May 13;7:710. doi: 10.3389/fpsyg.2016.00710 (PMC4865493; doi:10.3389/fpsyg.2016.00710)
Supplement: Supplementary file 1 [file Table_1.DOCX]

Appendix A

*The eight semantic categories and 64 target items (with frequency counts) used in Experiments 1-3.*

|  |  |  |  |  |  |  |  |
| --- | --- | --- | --- | --- | --- | --- | --- |
| High-frequency | | | |  | Low-frequency | | |
| Category | | Item | Frequency |  | Category | Item | Frequency |
| Animal | | Bear | 57 |  | Utensils | Fork | 14 |
|  |  | Cat | 23 |  |  | Jar | 16 |
|  |  | Dog | 75 |  |  | Plate | 22 |
|  |  | Sheep | 23 |  |  | Tray | 18 |
|  |  | Lion | 17 |  |  | Pan | 16 |
|  |  | Cow | 29 |  |  | Mug | 1 |
|  |  | Rabbit | 11 |  |  | Spoon | 6 |
|  |  | Horse | 117 |  |  | Bowl | 23 |
| Clothing | | Shoe | 14 |  | Tools | Hammer | 9 |
|  |  | Dress | 67 |  |  | Pliers | 1 |
|  |  | Belt | 29 |  |  | Flashlight | 8 |
|  |  | Hat | 56 |  |  | Axe | 6 |
|  |  | Shirt | 27 |  |  | Ladder | 19 |
|  |  | Watch | 81 |  |  | Rope | 15 |
|  |  | Jacket | 33 |  |  | Nail | 6 |
|  |  | Tie | 23 |  |  | Chisel | 4 |
| Vehicles | | Car | 274 |  | Instruments | Cello | 0 |
|  |  | Tractor | 24 |  |  | Guitar | 19 |
|  |  | Boat | 72 |  |  | Flute | 1 |
|  |  | Airplane | 114 |  |  | Organ | 12 |
|  |  | Van | 32 |  |  | Trumpet | 7 |
|  |  | Bus | 34 |  |  | Banjo | 2 |
|  |  | Wagon | 55 |  |  | Drum | 11 |
|  |  | Train | 82 |  |  | Harp | 1 |
| Furniture | | Chair | 66 |  | Birds | Crow | 2 |
|  |  | Bed | 127 |  |  | Eagle | 5 |
|  |  | Table | 198 |  |  | Ostrich | 0 |
|  |  | Couch | 12 |  |  | Penguin | 0 |
|  |  | Rug | 13 |  |  | Duck | 9 |
|  |  | Desk | 65 |  |  | Pigeon | 3 |
|  |  | Bureau | 43 |  |  | Owl | 2 |
|  |  | Lamp | 18 |  |  | Hen | 22 |
|  | | **Mean** | 59.72 |  |  | **Mean** | 8.75 |
